# The roles of tumor-derived exosomes in non-small cell lung cancer and their clinical implications

**DOI:** 10.1186/s13046-018-0901-5

**Published:** 2018-09-14

**Authors:** Hongmei Zheng, Yuting Zhan, Sile Liu, Junmi Lu, Jiadi Luo, Juan Feng, Songqing Fan

**Affiliations:** Department of Pathology, The Second Xiangya Hospital, Central South University, Changsha, 410011 Hunan China

**Keywords:** NSCLC, Tumor-derived exosomes, Clinical implications, Targeted therapy

## Abstract

Non-small cell lung cancer (NSCLC) accounts for approximately 85% of lung cancer cases, and it is one of the leading causes of cancer death in both men and women worldwide due to diagnosis in the advanced stage, rapid metastasis, and recurrence. At present, precision molecular targeted therapeutics directed toward NSCLC driven genes has made great progress and significantly improved the overall survival of patients with NSCLC, but can easily lead to acquired drug resistance. New methods are needed to develop real-time monitoring of drug efficacy and drug resistance, such as new molecular markers for more effective early detection and prediction of prognosis. Exosomes are nano-sized extracellular vesicles, containing proteins, nucleic acids and lipids, which are secreted by various cells, and they play an important role in the development of lung cancer by controlling a wide range of pathways. Tumor-derived exosomes are of great significance for guiding the targeted therapy of NSCLC and exosomes themselves can be a target for treatment. In this review, we describe the potential roles of tumor-derived exosomes and their clinical significance in NSCLC.

## Background

Lung cancer is one of the leading causes of cancer-related death both in men and women [1] and remains the most frequently diagnosed cancer in the world [2], which is classified into two histological subtypes: non-small cell lung cancer(NSCLC)accounting for 85% and small-cell lung cancer (SCLC) accounting for the remaining 15% [3]. Only 17.7% of the patients live over 5 years after being diagnosed with lung cancer [4]. The standard treatment of NSCLC is curative surgical resection, combined with or without chemoradiotherapy for the patients with early-stage including stage I, II and a part of stage III [5, 6]. However, most patients with NSCLC are diagnosed in the advanced stage [7], which makes the cancer difficult to surgically resect and increases the rate of postoperative recurrence, while the effect of radiotherapy and chemotherapy has plateaued [8]. On the other hand, treatment of NSCLC is evolving from the use of cytotoxic chemotherapy to precision treatment based on changes in molecular and gene levels [9], which inevitably leads to drug resistance sooner or later. In the past, the golden standard of the patients’ diagnosis and gene mutations detection is tissue biopsy, which limits the assessment of lung cancer development and prognosis due to tumor heterogeneity and evolution [10]. Liquid biopsy is typically used to separate and analyze circulating free DNA and RNA from the blood of cancer patients, or other body fluids, which has potential advantages, such as real-time monitoring of treatment response, rapid and accurate identification of drug resistance genes, identification of minimal residual disease and prediction of prognosis [10, 11]. Exosomes are nano-sized extracellular vesicles, containing proteins, nucleic acids and lipids and the encapsulated contents in exosomes can escape from the degradation. Exosomes play an important role in cell-to-cell communication, tumor progression and drug resistance and have excellent prospects in liquid biopsy [12–14]. In this review, we discuss the close relationship between tumor-derived exosomes and NSCLC, and the application of tumor-derived exosomes in the targeted therapy for NSCLC.

### Exosomes and tumor-derived exosomes

**Fig. 1:**
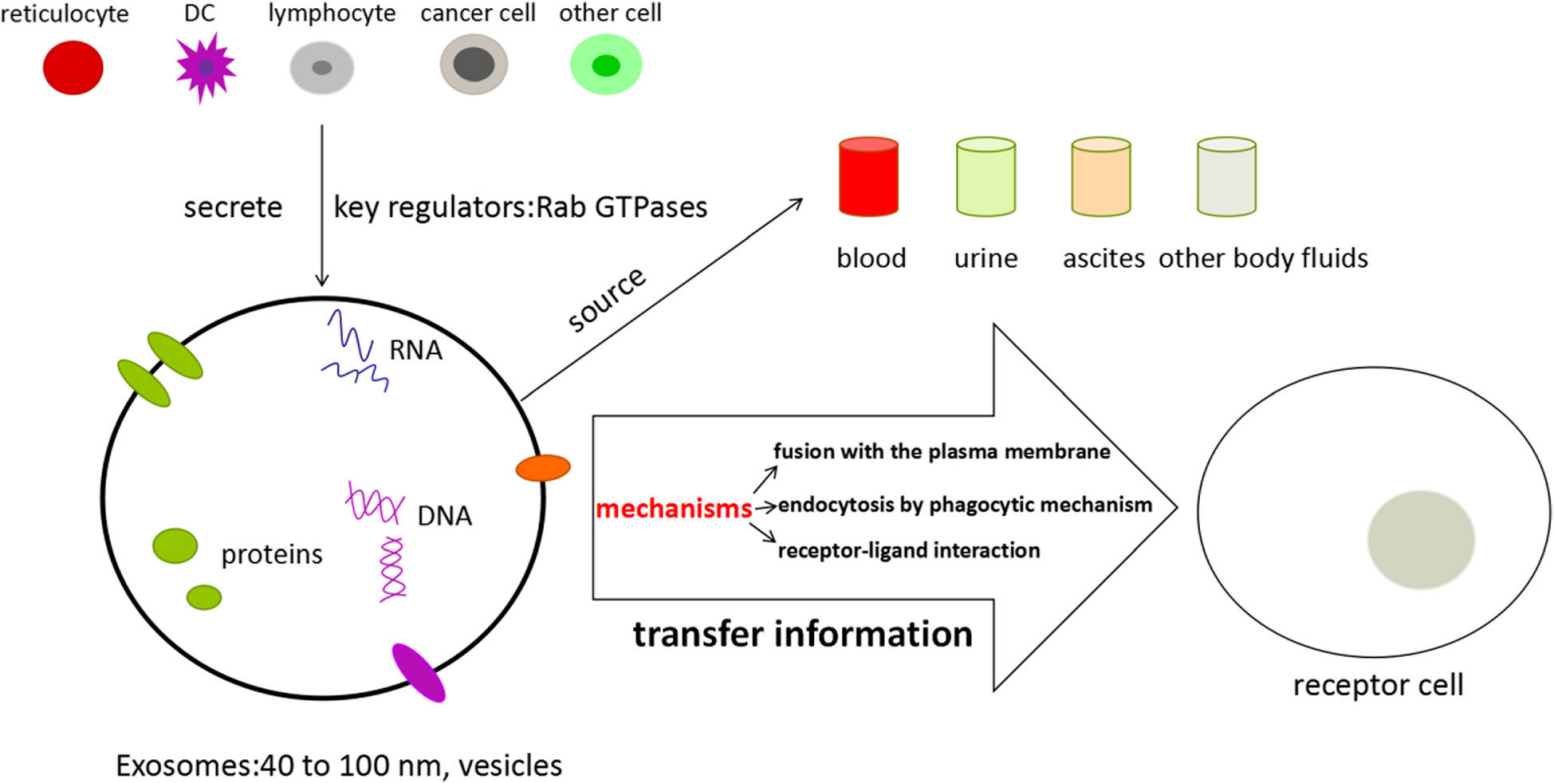
The biology of exosomes**.** Exosomes are some vesicles ranged from 40 to 100 nm, which can be released by many cells and purified from many body fluids. Exosomes can transfer information to the receptor cells through fusion with the plasma membrane, endocytosis by phagocytic mechanism or receptor-ligand interaction with the cell

The diameter of exosomes which was first reported in the process of sheep reticulocytes maturation ranged from 40 to 100 nm, showing a characteristic cup-shaped morphology (after negative staining) or round well-delimited vesicles observed by transmission and cryo-electron microscope [15, 16]. Exosomes are small vesicles of endosomes that can be released by many cell types, such as reticulocytes [16], dendritic cells [17], lymphocytes [18, 19] and cancer cells [20]. Furthermore, exosomes have been successfully purified from many body fluids such as blood, urine, pleural effusions, ascites and broncoalveolar fluid [21]. Also, exosomes can transfer information, including DNA, RNA and proteins, to the receptor cells through fusion with the plasma membrane, endocytosis by phagocytic mechanism or receptor-ligand interaction with the cell [22] and have the heterogeneous biological behaviors due to their different secretory cell types with diverse cell status [23], thus participating in different physiological and pathological processes. Rab GTPases, including Rab27a and Rab27b, are the key regulators of exosomes secretion and Rab27 is closely related to the occurrence and the development of tumor, which indicates the role of exosomes secretion in tumor biology [24]. (These are summarized in Fig. 1).

The immunological activities of exosomes affect the mechanisms of immunoregulation including immune activation, antigen expression regulation, intercellular communication, immunization surveillance, and immunosuppression [25]. Tumor-derived exosomes which carry tumor-associated antigens can interfere with anti-tumor immunotherapies [26]. It has been reported that about 2000 trillion exosomes can be detected in normal human blood versus 4000 trillion exosomes in the blood of cancer patients [27], which indicates that tumor cells produce more exosomes than their normal counterparts and can be a useful diagnostic biomarker [28]. Tumor-derived exosomes are believed to be important mediators of intercellular signaling and epithelial–mesenchymal transition (EMT), which transforms cancer cells into more aggressive phenotype, and contributes to the tropism of metastatic disease in specific cancer types with pre-metastatic niche [29]. Azmi et al. found tumor cells exposed to hypoxia secreted exosomes which had enhanced potential of angiogenesis and metastasis, suggesting that tumor cells adapt themselves to the hypoxic microenvironment by secreting exosomes, so as to stimulate angiogenesis or create a more favorable tumor microenvironment to promote tumor metastasis [30]. Furthermore, tumor-derived exosomes are of crucial importance in tumor growth and drug resistance, as they transfer nucleic acids and oncogenic proteins to the tumor cells, which indicates tumor-derived exosomes and their contents may be of potential value in the diagnosis, prognosis, prediction of treatment response and targeted therapy [31]. Due to the importance of tumor-derived exosomes, the methods to detect exosomes including isolation purification and analysis require further development (These methods are summarized in Table 1).

**Table 1:** Detection methods of exosomes

Contents	Methods	References
Isolation and purification	Ultracentrifugation Density gradients centrifugation Immunobeads Size exclusion chromatography Spin column-based methods	[127–131]
Analysis of size and shape	TEM	[132]
Analysis of size distribution and concentration	Nanoparticle Tracking Analysis FAVS	[133–135]
Analysis of proteins	Mass spectrometry, Western-blot SDS-PAGE	[136, 137]
Analysis of RNA	NGS, Microarray, RT-PCR	[138–140]

### Roles of Tumor-derived exosomes in the NSCLC

**Fig. 2:**
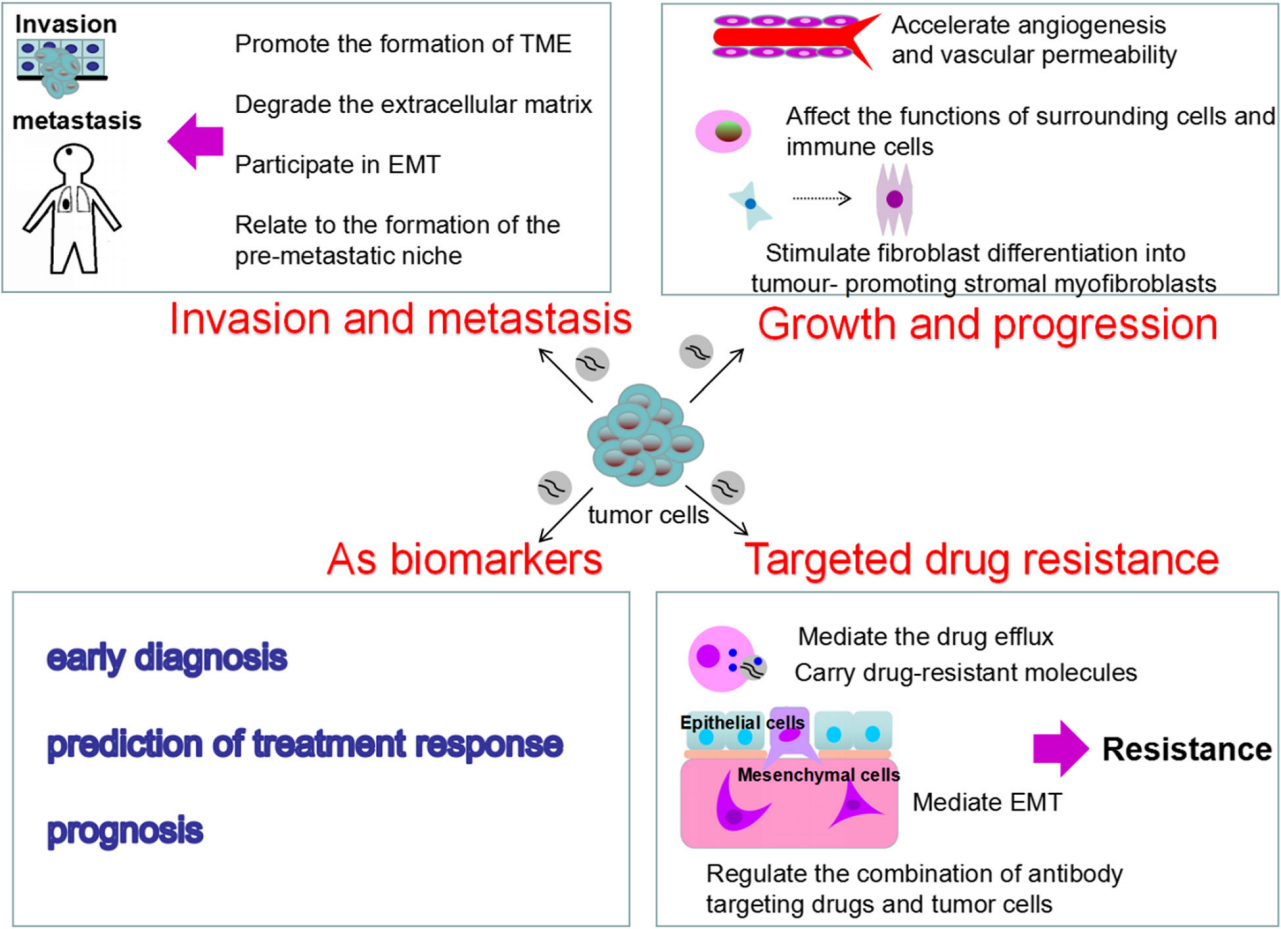
Roles of Tumor-derived exosomes in the NSCLC. Tumor-derived exosomes play a vital role in tumor growth and progression, invasion and metastasis, targeted drug resistance, and can also be used as biomarkers for early diagnosis, prediction of treatment response and prognosis

The formation and development of NSCLC is influenced by many factors and mechanisms, which is a long-term and complex process. Exosomes secreted by lung cancer cells play a vital role in this process as mediators of intercellular communication. (These roles are summarized in Fig. 2).

### Tumor-derived exosomes in the growth and progression of NSCLC

It has been shown that cancer-associated fibroblasts (CAFs) in the tumor microenvironment can secrete exosomes containing lipids, amino acids, and TCA-cycle intermediates which can promote tumor growth under nutrient-stressed or nutrient-deprivation conditions [32]. Angiogenesis is also vital for tumor growth since tumor vessels are the important sources of nutrient substances in the tumor cells [33]. Tumor-derived exosomes can accelerate angiogenesis and tumor growth in a TGFβ1 (transforming growth factor β1)-dependent pathway to stimulate fibroblasts to differentiate into tumor-promoting stromal myofibroblasts [34]. Exosomal miR-23a from hypoxic lung cancer cells can increase angiogenesis and vascular permeability by targeting tight junction protein ZO-1(zonula occludens-1) and prolyl hydroxylase [35]. STAT3-regulated exosomal miR-21 enhances the level of vascular endothelial growth factor (VEGF), thereby promoting tumor angiogenesis and inducing malignant transformation of bronchial epithelial cells [36]. Exosomal miR-210 from lung cancer regulates the level of tyrosine receptor kinase A3 (ephrin A3) in matrix cells and promotes tumor angiogenesis to maintain the growth of tumor cells [37]. All of these indicated that tumor-derived exosomes can promote the growth and progression of NSCLC by angiogenesis. Lung cancer cell-derived exosomes can also affect the progression of lung cancer by affecting the physiological functions of other cells in the surrounding tissues and microenvironment. Mesenchymal stem cell (MSC) is an important one among these cells, which can be transformed into a pro-inflammatory phenotype via NFκB-TLR signaling pathway regulated by lung cancer cell-derived exosomes, thus promoting the growth of lung cancer [38]. Furthermore, lung cancer cell-derived exosomes can indirectly regulate the progression of tumor by affecting the function of immune cells in the tumor microenvironment. Tumor-derived exosomes can help with immune escape by transferring specific proteins and RNA into the receptor cells [39] and promote tumor progression by reprogramming functions of immune cells [26]. Lung cancer cell-derived exosomes containing the epidermal growth factor receptor (EGFR) can induce tolerogenic dendritic cells and tumor antigen-specific regulatory T cells (Treg) which can inhibit the function of CD positive T cells with anti-tumor function and promote the growth of lung cancer [40, 41]. Lung cancer cell-derived exosomes containing HSP72 (heat shock protein 72) can activate the STAT3 signaling pathway to mediate the immunosuppressive effect of MDSCs (myeloid-derived suppressor cells) and thereby suppress T cell activation [42, 43]. In conclusion, these studies revealed that tumor-derived exosomes could play an important role in the growth and progression of NSCLC with different contents and provide a novel future for the therapy of NSCLC, which should be further researched.

### Tumor-derived exosomes in the invasion and metastasis of NSCLC

Metastasis is one of the major causes of death in lung cancer patients, which is related to a variety of mechanisms and involves multiple steps [44]. Tumor-derived exosomes, as carriers of information transmission, promote metastasis through their direct or indirect roles [13]. Exosomes can promote the formation of the lung cancer microenvironment to increase the invasiveness of tumor cells [45]. Due to the instability of oncogenes, hypoxia, acidosis and inflammatory immune response can promote tumor cells to release more exosomes to form tumor microenvironment [46, 47] which is beneficial to the rapid growth of tumor cells and enhances their ability of invasion. Tumor-derived exosomes are associated with invadopodia that initiates invasion through degradation of the extracellular matrix [48]. Exosomal contents can also promote metastasis and transfer metastatic potential to recipient cells [13]. EMT is the disappearance of the epithelial-like characteristics and gains the phenotype of stromal cells, [49] which is an important process before the metastasis of the tumor cells and also a complex process, including cytoskeleton changes, down-regulation of the expression of adherens junction molecule E-cadherin and so on [50]. It has been reported that several proteins and miRNAs are involved in the EMT [51–54]. Tumor-derived exosomes have also been reported to be associated with the formation of the pre-metastatic niche [13] that forms at the site of the future metastasis and promotes the growth of disseminated tumor cells [55]. The main sites of NSCLC metastasis are brain, adrenal gland, bone and the liver [44]. Different types of metastatic cancer cells have significant differences in organ tropism [56] which is associated with tumor-derived exosomal integrins [57]. (The major findings of tumor-derived exosomes associated with invasion and metastasis in NSCLC are summarized in Table 2).

**Table 2:** Major findings of tumor-derived exosomes in NSCLC

Contents	Findings	References
Invasion	exosomal TGF-β and IL-10 may enhance migration ability in vitro under hypoxia	[47]
	exosomal vimentin may induce EMT	[51]
	exosomal Rab3D which activates AKT/GSK3β signaling may induce EMT	[52]
	exosomal miR-23a may affect the changes of EMT related phenotype	[53]
	Exosomal miR-302b can inhibit the proliferation and migration of lung cancer cells through the TGFβRII/ERK pathway	[54]
Drug resistance	exosomal VEGF and TGF2β may result in drug resistance	[61, 62]
	exosomal proteins and phospholipids components are involved in gefitinib resistance	[63, 64]
	exosomes may antagonize the chemotherapeutic effect of cisplatin by upregulating autophagy	[65]
	exosomes can regulate the combination of antibody targeting drugs and tumor cells	[71, 72]

### Tumor-derived exosomes in the targeted drug resistance

Resistance to chemotherapy, radiotherapy and targeted therapy remains a major obstacle to cancer treatment [30]. Drug resistance is a multifaceted problem. It has already been reported that resistance of targeted therapy and platinum based chemotherapy is associated with microRNAs [58–60]. Here the authors discuss mechanisms associated with exosomes. (1) Tumor-derived exosomes mediate EMT by transferring related tissue factors (such as VEGF, TGF2β), and thus the tumor cells can resist apoptosis, which usually result in drug resistance [61, 62]. (2) Tumor cells and stromal cells in the tumor microenvironment can secrete exosomes carrying drug-resistant molecules (miRNAs, proteins) which are transferred to enhance the tolerance of the tumor cells to the drug through the interaction of exosomes in the TME (tumor microenvironment) [63–65]. (3) Tumor-derived exosomes can also mediate the drug efflux by transferring multi-drug resistant (MDR) protein encapsulated drug, thus influencing the drug efficacy. In the development of many malignant tumors, a special transporter system which is MDR-associated ATP-binding cassette transporter (MDR-ABC) is activated to mediate the drug from the intracellular to the extracellular [66]. MDR-ABC is a type of intracellular transporter proteins, which has the same transmembrane domain and is usually located on the membrane of exosomes and multivesicular bodies (MVBs). The chemotherapeutic or targeted therapy drug and its metabolites in the cell can be transferred into the inner body which can aggregate to form MVBs. When the MVBs are fused with the cell membrane, the inner body is released into the extracellular matrix in the form of exosomes and completes the drug efflux [67–71]. (4) Tumor-derived exosomes can also influence the drug effect by regulating the combination of antibody targeting drugs and tumor cells to enhance the drug resistance of tumor cells [71, 72]. All of these mechanisms associated with exosomes can induce the targeted therapy drug resistance. (The major findings of tumor-derived exosomes associated with drug resistance in NSCLC are summarized in Table 2).

### Tumor-derived exosomes as markers in prognosis of NSCLC

Increasingly, studies suggest that exosomal miRNAs and proteins are positively associated with the stage and degree of tumor progression [73], indicating that exosomal components can be used as prognostic markers to improve treatment selection for the patients with NSCLC [74]. For example, Sandfeld-Paulsen et al. investigated exosomes from plasma of 276 non-small-cell lung cancer patients, which were phenotyped by using the Extracellular Vesicle Array. As a result, they found that NY-ESO-1, PLAP, EGFR, Alix and EpCam were correlated to overall survival (OS), which indicated that exosomal membrane-bound proteins were strong prognostic biomarkers in NSCLC [74]. In addition, Liu et al. found that elevated levels of exosomal miR-10b-5p, miR-23b-3p and miR-21-5p were associated with poor overall survival by analyzing 84 plasma exosomal miRNAs in lung adenocarcinoma patients and healthy controls, which suggested that these exosomal miRNAs may also be used as prognostic biomarkers of NSCLC [75]. The downregulation of the miRNA-146-5p indicated a poor progression free survival (PFS) compared to patients with higher exosomal miRNA [76]. All of these indicate that exosomes can be used as non-invasive prognostic biomarkers.

### Clinical implications of tumor-derived exosomes in the targeted therapy of NSCLC

Liquid biopsy samples are becoming more frequently used in blood or other body fluids as biomarkers for NSCLC early diagnosis, treatment guidance and drug resistance monitoring [77]. The specificity of the cell types and the accessibility from the body fluids make the exosomes important candidates for the diagnosis and target therapy of tumors [78].

### Tumor-derived exosomes as biomarkers for NSCLC diagnosis and targeted therapy guidance

The exosomes secreted by lung cancer cells, which enrich various proteins, such as EGFR, KRAS, claudins and RAB-family proteins, and promote the development of lung cancer, are effective biomarkers for early diagnosis of lung cancer [79] and the basis of targeted therapy. For example, Birgitte et al. used EV array to detect exosomal proteins in NSCLC tissues and normal tissues, and found that markers CD151, CD171 and tetraspanin 8 were higher in patients with cancer of all histological subtypes than patients without cancer [80]. Huang and colleagues carried out immunostaining analysis of exosomes of NSCLC tissues and chronic pneumonia tissues, and found that 80% of the NSCLC specimens were EGFR positive on the surface of the exosomes, while only 2% of the chronic pneumonic tissues were EGFR positive, which suggested that exosomal EGFR protein could be used as a biomarker for differential diagnosis of lung cancer [41] and indicated that gene detection could be further carried out to provide a molecular basis for targeted therapy. Recently, the translocation ALK-EML4 has also been identified inside the exosomes [81], which is a biomarker for response to first generation ALK-TKIs [82]. Vykoukal and colleagues found that the expression of 108 proteins in vesicle preparations of lung adenocarcinoma plasmas was significantly different compared to controls, of which 43 were identified in EVs from lung adenocarcinoma cell lines [83].

MicroRNAs (MiRNAs) are a class of small noncoding RNAs (the length of 18–25 nucleotides), post-transcriptional regulation molecules expressed in many organisms [84]. The expression of miRNAs, like other cancer-associated genes expression, can be altered by chromosomal amplification/deletion, transcription factor activation [85]. It has been found that the miRNAs expression profiles of NSCLC exosomes which are often detected by miRNA-microarray are different from normal tissues. Zhao et al. [86] analyzed the expression of the plasma exosomal miRNAs in the tumor tissues of 150 patients with non-small cell lung cancer, and found that plasma levels of the exosomal hsa-miRNAs (hsa-miR-25, hsa-miR-122, hsa-miR-195, hsa-miR-21 and hsa-miR-125b) were associated with EGFR mutation, which could help to determine whether or not to use targeted therapy drugs and provide a new way to detect NSCLC gene mutations. The miRNAs for adenocarcinoma diagnosis showed an AUC value of 0.936, with a sensitivity of 80.65% and a specificity of 91.67% [87]. Two studies showed that upregulation of miR-1246 and miR-208-a was associated with resistance to radiotherapy and high proliferation of the tumor by targeting the genes p21 and DR5 respectively, which could not only lead to a prognostic biomarker, but also to a new target against NSCLC [88]. It has been possible to detect the rearrangement of EML4-ALK in NSCLC patients by analyzing the exosomal miRNAs in blood and has been proved that the anaplastic lymphoma kinase (ALK)-EML4 translocation inside the exosomes with a specificity of 100% and a sensitivity of 64% [89].

In summary, exosomes are becoming increasingly important in the diagnosis of NSCLC as biomarkers and the molecular basis of NSCLC targeted therapy.

### Tumor-derived exosomes as biomarkers for NSCLC targeted therapy resistance

With the increasing understanding of molecular biology and genetics of tumors, the research and clinical application of targeted therapy has become a hot topic, which can improve prognosis and also guide therapeutic decision, thus reducing morbidity and mortality. On average, there are more than 300 mutations in each lung cancer, but only a few of these genes can promote or “drive” the lung tumorigenesis [90], mainly including EGFR_(epidermal growth factor receptor), ALK_(anaplastic lymphoma kinase), c-met and so on [91–93]. EGFR, also called ErbB1 or HER1, is involved in the signal transduction pathway of cell proliferation and apoptosis, which is a part of the ErbB family of transmembrane receptor tyrosine kinases [94]. The EGFR mutations, first reported in 2004 [95], usually contain in-frame duplications/insertions in exon 20, in-frame deletions (around amino acid residues 747 to 750) in exon 19 and single missense mutations in exon 21_(L858R mutation) [96], and reveal a potential responsiveness of NSCLC to the tyrosine kinase inhibitors [97]. EGFR TKIs improve time to progression, response rates and overall survival, but acquired resistance to EGFR-TKIs is inevitable [98], and thereby tools to predict the risk of drug resistance are necessary to improve the clinical treatment choices. Tumor-derived exosomes can be used as biomarkers to evaluate therapeutic effect of targeted therapy by liquid biopsy. T790 M mutation is found in patients treated with EGFR-TKIs [99], accounting for about 50–60% with acquired resistance to erlotinib or gefitinib [99, 100]. The third generation of EGFR-TKIs includes osimertinib which overcomes T790 M-mediated resistance to EGFR-TKIs [101], but it’s still unable to avoid the targeted therapy drug resistance, and some of the patients develop a new drug resistance mutation C797S [102]. It has been reported that exosomal RNA is used to detect EGFR T790 M and activating EGFR mutations, and the sensitivity is 90% and 98%, respectively [103]. So it’s possible to identify targeted therapy drug resistance in the tumor-derived exosomes. In addition, Choi et al. [63]. identified PC9R cells with rich EV proteins were resistant to gefitinib due to EGFR T790 M mutation by Nano-LC–MS/MS analysis and would help to develop new diagnostic strategies to predict and assess the drug resistance of gefitinib. Chromosome rearrangements of ALK are detected in the NSCLC of 3–7%, which indicates a response to first generation ALK-TKIs (such as crizotinib). However, most patients develop resistance to this therapy [104]. L1196 M and G1269A are identified as secondary mutation, accounting for resistance to ALK-TKIs [104, 105]. This acquired drug resistance may be predicted by detecting exosomes.

### The potential value of tumor-derived exosomes in the targeted therapy of NSCLC

Studies have found that research based on exosomes and related components can provide new inspiration for the precision treatment of NSCLC.

Exosomes have been developed as drug delivery vehicles for a variety of drugs, such as small molecular drugs, nucleic acid proteins and other drugs for cancer treatment, with low immunogenicity and toxicity [106, 107]. Lai et al. used electroporation or lipofection to transfect the drugs of interest directly into the exosomes or transfer the genes that encode for the RNA/protein of interest into exosome-secreting cells [108]. Mendt and colleagues established a standard operating procedure to generate engineered exosomes which had the ability to target oncogenic Kras (iExosomes), and was confirmed to suppress oncogenic Kras and increase the survival of mouse models with pancreatic cancer, which laid the foundation for exosomes in the targeted therapy of NSCLC [109]. Exosomes can be targetable to specific tissues, resistant to metabolic processes and membrane-permeable [110], which have a wide application prospect in the targeted therapy of NSCLC.

Exosomes play an important role in tumor development, which indicates reducing the contents of exosomes can help the targeted therapy of NSCLC. Exosomes may also serve as a direct target for NSCLC. Data shows that the prevention of exosomes production can inhibit tumorigenesis and a series of methods have been suggested to reduce the contents of exosomes [110]: (1) Blood purification: Aethlon Medical designed a blood filtration therapy which could capture a large number of antibodies and other similar substances, such as aptamers, protein ligands, and exosomes to realize the new treatment of NSCLC [111]. Other studies have shown that the use of hollow fiber filtration technology to remove exosomes from the patient’s blood can minimize the immune tolerance caused by the exosomes [112]. (2) Change the local pH of the tumor: In addition to blood purification, proton pump inhibitors (PPIs) can also improve the low pH of cells by PPI pretreatment in vivo to reduce the contents of tumor-derived exosomes in the plasma, so PPI may likely be an effective method to inhibit the secretion of exosomes in NSCLC [113]. (3) Drug usage: For example, Fabbri et al. [114]. found that the use of GW4869, a neutral sphingomyelase inhibitor (regulates ceramide biosynthesis, promotes exosomes inward budding), could inhibit the production of exosomes in mice and reduce the metastasis of lung cancer. Some studies directly target exosomes as a drug target. For example, amiloride can inhibit the synthesis and secretion of exosomes [115], and diannexin can impede the absorption of exosomes by receptor cells [116]. (4) Interfere with signal pathway: some studies have shown that interference with the signal pathway associated with production or secretion of tumor-derived exosomes can inhibit the exosomes secretion. Ostrowski and colleagues found that knockout Rab27 or its effector (SYTL4 and EXPH5) could inhibit the secretion of exosomes in HeLa cells [24]. It may be useful for inhibiting the secretion of exosomes in NSCLC. It has already been proved that ISGylation as a novel ubiquitin-like modifier can control exosomes production [117] and syndecan-syntenin-ALIX plays a key role in the biogenesis of exosomes [118], which can also be targeted in NSCLC.

The diversity of exosomes components and functions associated with NSCLC provides multiple potential therapeutic targets for the treatment of NSCLC. Yang et al. found that the promotion of the expression of let-7 in exosomes was a potential target for the treatment of NSCLC because of its high tumor suppressor effect, great clinical significance and tissue specificity [119]. Zhang et al. [120]. identified that exosomes separated from H460 cells with restoration of LKB1 (liver kinase B1) had a higher ability in lung cancer cell migration, which could be a novel target. Nao et al. [121]. developed a new strategy of antibody therapy with anti-CD9 or anti-CD63 to target on tumor-derived exosomes and inhibit metastasis of breast cancer in mouse models, which laid the foundation for targeted therapy of NSCLC. Lung cancer cells can secrete survivin through exosomes to inhibit the apoptosis of lung cancer cells and promote the growth of lung cancer cells, so the use of survivin gene negative mutant (Survivin-D53A) can promote the apoptosis of NSCLC cells and may become a potential gene therapy drug [122–124]. Previously described exosomal miR-302b also provided a potential target for the treatment of NSCLC [54]. The exosomes membrane can contain specific tumor antigen through gene transformation, which has a certain target function and can be used for the treatment of NSCLC [125, 126].

In summary, studies on the treatment of lung cancer-related exosomes will provide a new idea for exploring precision treatment of NSCLC. (These potential values are summarized in Table 3).

**Table 3:** Tumor-derived exosomes in the targeted therapy of NSCLC

Potential value	Methods	References
A tool for drug delivery	Use electroporation or lipofection	[106–110]
A direct target for reducing the content	Blood purification: ADAPT, hollow fiber filtration technology Change the local pH of the tumor: PPIs Drug usage: GW4869, amiloride, diannexin Interfere with signal pathway: knockout Rab27, SYTL4 ,EXPH5, ISGylation and syndecan-syntenin-ALIX	[24, 110–118]
Other targets	let-7, LKB1, CD9, CD63, surviving, miR-302b	[54, 119–126]

## Conclusion

In 2006, WHO officially identified lung cancer as a chronic controlled disease whose occurrence and development was a multidirectional, multistep complex network process. Liquid biopsy for tumor-derived exosomes has the advantages of noninvasive and real-time monitoring, which provides a new reference for precision medical and individualized treatment, and develops a new method for early diagnosis, prognosis evaluation, drug delivery and targeted therapy. At present, the application of exosomes in the diagnosis and treatment of NSCLC is still in their initial stage. In the future, further study in exosomes, including biogenesis, secretion, interaction with target cells and the roles of exosomal components may improve applications to medical treatment and improve the survival rate of NSCLC patients. A variety of problems remain to be overcame: (1) The specific mechanism of exosomes as an important part of the tumor microenvironment in the evolution of the NSCLC is not yet clear; (2) The sensitivity and specificity of exosomes application in the diagnosis and treatment of NSCLC still need improvement; (3) The acquisition of high purity exosomes is still an issue due to technical limitation and high cost; (4) The quantification, purification and preservation of exosomes has not yet been standardized; (5) The side effects of exosomes used in the targeted therapy cannot be completely determined. All of these problems restrict the application of exosomes in NSCLC. These reviews synthetically expound the multi-faceted nature of exosomes and potential value in the targeted therapy of NSCLC.

## Abbreviations

ADAPT: Adaptive dialysis-like affinity platform technology
AKT: Protein Kinase B
ALK: Anaplastic lymphoma kinase
CAFs: Cancer-associated fibroblasts
EGFR: Epidermal growth factor receptor
EMT: Epithelial–mesenchymal-transition
ERK: Extracellular signal-regulated kinase
GSK3β: Glycogen synthase kinase 3β
GTPase: Guanosine triphosphatase
HSP72: Heat shock protein 72
IL-10: Interleukin-10
KRAS: Kirsten rat sarcoma viral oncogene
LKB1: Liver kinase B1
MDR: Multi-drug resistant
MDR-ABC: MDR-associated ATP-binding cassette transporter
MDSCs: Myeloid-derived suppressor cells
MSCs: Mesenchymal stem cells
MVBs: Multivesicular bodies
NF-kB: Nuclear factor kappa B
NSCLC: Non-small cell lung cancer
OS: Overall survival
PFS: Progression free survival
PPIs: Proton pump inhibitors
SCLC: Small-cell lung cancer
STAT3: Signal transducers and activators of transcription 3
TD-exosomes: Tumor-derived exosomes
TGF-β: Transforming growth factor β
TGF-β1: Transforming growth factor β1
TKIs: Tyrosine kinase inhibitors
TLR: Toll-like receptor
TME: Tumor microenvironment
Treg: Regulatory T cell
VEGF: Vascular endothelial growth factor
ZO-1: Zonula occludens-1
